# Associations Between HbA1c Across the Normal Range, Diagnosed, and Undiagnosed Diabetes and Retinal Layer Thickness in UK Biobank Cohort

**DOI:** 10.1167/tvst.12.2.25

**Published:** 2023-02-16

**Authors:** Sharon Y. L. Chua, Paul Welsh, Zihan Sun, Konstantinos Balaskas, Alasdair Warwick, David Steel, Sobha Sivaprasad, Roomasa Channa, Tony Ko, Naveed Sattar, Anthony P. Khawaja, Paul J. Foster, Praveen J. Patel, on behalf of The UK Biobank Eye and Vision Consortium

**Affiliations:** 1 NIHR Biomedical Research Centre at Moorfields Eye Hospital NHS Foundation Trust & UCL Institute of Ophthalmology, London, UK; 2 Institute of Cardiovascular & Medical Sciences, University of Glasgow, Glasgow, UK; 3 School of Biological Sciences, University of Manchester, Manchester, UK; 4 UCL Institute of Cardiovascular Science, University College London, London, UK; 5 Sunderland Eye Infirmary, Sunderland, UK; 6 Bioscience Institute, Newcastle University, Newcastle Upon Tyne, UK; 7 Department of Ophthalmology, University of Wisconsin - Madison, Madison, WI, USA; 8 Topcon Healthcare Solutions Research & Development, Oakland, NJ, USA

**Keywords:** diabetes, HbA1c, optical coherence tomography (OCT), retinal layer thickness, photoreceptor layer

## Abstract

**Purpose:**

The purpose of this study was to investigate the association between glycated hemoglobin (HbA1c) levels and retinal sub-layer thicknesses in people with and without diabetes.

**Methods:**

We included 41,453 UK Biobank participants aged 40 to 69 years old. Diabetes status was defined by self-report of diagnosis or use of insulin. Participants were categorized into groups: (1) those with HbA1c <48 mmol/mol were subdivided into quintiles according to normal range of HbA1c; (2) those previously diagnosed with diabetes with no evidence of diabetic retinopathy; and (3) undiagnosed diabetes: >48 mmol/mol. Total macular and retinal sub-layer thicknesses were derived from spectral-domain optical coherence tomography (SD-OCT) images. Multivariable linear regression was used to evaluate the associations between diabetes status and retinal layer thickness.

**Results:**

Compared with participants in the second quintile of the normal HbA1c range, those in the fifth quintile had a thinner photoreceptor layer thickness (−0.33 µm, *P* = 0.006). Participants with diagnosed diabetes had a thinner macular retinal nerve fiber layer (mRNFL; −0.58 µm, *P* < 0.001), photoreceptor layer thickness (−0.94 µm, *P* < 0.001), and total macular thickness (−1.61 µm, *P* < 0.001), whereas undiagnosed diabetes participants had a reduced photoreceptor layer thickness (−1.22 µm, *P* = 0.009) and total macular thickness (−2.26 µm, *P* = 0.005). Compared to participants without diabetes, those with diabetes had a thinner mRNFL (−0.50 µm, *P* < 0.001), photoreceptor layer thickness (−0.77 µm, *P* < 0.001), and total macular thickness (−1.36 µm, *P* < 0.001).

**Conclusions:**

Participants with higher HbA1c in the normal range had marginally thinner photoreceptor thickness, whereas those with diabetes (including undiagnosed diabetes) had meaningfully thinner retinal sublayer and total macular thickness.

**Translational Relevance:**

We showed that early retinal neurodegeneration occurs in people whose HbA1c levels are below the current diabetes diagnostic threshold; this might impact the management of pre-diabetes individuals.

## Introduction

In the last decade, the concept of diabetic retinopathy (DR) as a microvascular disease has evolved. It is now considered a more complex complication in which neurodegeneration plays a significant role.^1^^–^^3^ In fact, the American Diabetes Association (ADA) has recently defined DR as a highly tissue-specific neurovascular complication involving progressive disruption of the interdependence between multiple retinal cell types.^4^

Technological advances in retinal imaging allow unprecedented resolution of retinal layer structure through spectral-domain optical coherence tomography (SD-OCT). This rapid, noninvasive, imaging modality uses optical reflectivity differences between different retinal layers to produce cross-sectional retinal images with a resolution of 4 to 6 µm. Thicknesses of each layer of retinal cells can be derived using automated retinal layer boundary detection and retinal layer segmentation.^5^ SD-OCT is extensively used to detect the evidence of retinal neurodegeneration in various sight-threatening diseases, such as glaucoma, optic neuropathies, and maculopathies.^6^^,^^7^

A growing body of evidence has shown that significant retinal thinning occurs in diabetic eyes without clinically detectable DR.^1^^,^^8^^,^^9^ This suggested that diabetic retinal neurodegeneration (DRN) may precede the microvascular changes of DR in patients with diabetes. However, little is known about whether this proceeds the diagnosis of diabetes. We hypothesized that in individuals within the normal range or with undiagnosed diabetes, higher HbA1c levels are associated with lower retinal thickness. Unlike previous studies, which examined the associations between glycated hemoglobin (HbA1c) levels and retinal layer thickness in people with diabetes,^10^^,^^11^ we examined the relationship between HbA1c and retinal layer thickness using the UK Biobank data resource, as well as comparing diabetic and non-diabetic participants. To the best of our knowledge, this is the first study to investigate this relationship among non-diabetic participants from the general community. We also looked at the association between diabetes status and retinal layer thickness.

## Methods

### Study Population

The UK Biobank is a very large community-based cohort of 502,656 UK residents registered with the National Health Service (NHS) and aged 40 to 69 years at enrollment. Briefly, baseline questionnaire, physical measurements, and biological samples were carried out between 2006 and 2010 at 22 study assessment centers. All participants gave written informed consent and the North West Multi-Center Research Ethics Committee approved the study in accordance with the principles of the Declaration of Helsinki. The overall study protocol (http://www.ukbiobank.ac.uk/resources/) and protocols for individual tests (http://biobank.ctsu.ox.ac.uk/crystal/docs.cgi) are available online. Participants answered a wide-ranging touch-screen questionnaire covering demographic, socioeconomic, lifestyle, systemic, and ocular diseases information. Ethnicity was classified into two categories (White versus non-White). The Townsend Deprivation Index was determined according to the participants’ postcodes at recruitment and the corresponding output areas from the preceding national census. The index was calculated based on the output area's employment status, home and car ownership, and household condition; the higher and more positive the index, the more deprived an area. Smoking status was classified into three categories (never, previous, and current). Physical measures included height and weight. Body mass index (BMI) was defined as weight divided by height squared. Blood pressure was measured twice, and the mean was used in the analysis. Antihypertensive medication was defined as self-reported use of medication to lower blood pressure. Definition of diabetes mellitus (DM) included self-reported diabetes and self-reported use of insulin. The definition of cardiovascular disease (CVD) included self-reported prior myocardial infarction, stroke, and transient ischemic attack as well as hospital diagnoses including the International Classification of Diseases (ICD)-10 codes I20 to 24, I63 to 64, and G45.

### Biochemistry Measurements

Biochemistry measures were performed at a central laboratory between 2014 and 2017. This included serum total cholesterol, high-density lipoprotein cholesterol (HDL-C), triglycerides (AU5400; Beckman Coulter), and HbA1c (VARIANT II TURBO Hemoglobin Testing System; Bio-Rad). Further details of these measurements and assay performances can be found in the UK Biobank online showcase and protocol.^12^

### Ocular Assessments

Ocular assessment was introduced as an enhancement in 2009 for 6 assessment centers which are spread across the United Kingdom.^13^ Refractive error was measured using an autorefractor (Tomey RC 5000, Nagoya, Japan) and spherical equivalent refraction (SER) was calculated as sphere power plus half cylinder power.^14^ Intraocular pressure was measured with the Ocular Response Analyzer (ORA; Reichert, Philadelphia, PA, USA) and included corneal-compensated intraocular pressure (IOP_cc_), which is thought to provide the most accurate assessment of the true IOP that is least affected by corneal properties.^15^ High resolution SD-OCT imaging was performed using the Topcon 3D OCT 1000 Mk2 (Topcon Inc, Oakland, NJ, USA) in a dark room, without pupillary dilation using the 3D macular volume scan (scan settings = 512 horizontal A scans per B scan; 128 B scans in a 6 × 6 mm raster pattern). The individual retinal surfaces were segmented using the Topcon Advanced Boundary Segmentation (TABS) algorithm (version 1.6.1.1).^16^ The TABS segmentation algorithm was used to segment the macular retinal nerve fiber layer (mRNFL), inner nuclear layer (INL), ganglion cell-inner plexiform layer (GCIPL), photoreceptor layer, and total macular thickness in a 6-mm diameter circle centered at the true fovea location, defined topographically across the macular by the Early Treatment Diabetic Retinopathy Study (ETDRS) grid.^17^ The average thickness parameters were derived from the TABS algorithm, of which the reproducibility had been assessed previously (intraclass correlation coefficients ranging from 0.954 to 0.933)^18^ We added the prefix “m” to the RNFL abbreviations to denote as macular measures, as the RNFL can be derived from images centered on the optic disc.

### Inclusion and Exclusion Criteria

We excluded participants who withdrew consent; who had self-reported ocular conditions, including diabetes-related eye disease, age-related macular degeneration (AMD), glaucoma, eye injury resulting in vision loss or other serious eye conditions; and high SER (<−6 diopters [D] or > +6 D). Participants diagnosed with diabetic retinopathy (ICD-10 code H360) were further excluded. Participants who had poor SD-OCT signal strength, image quality score <45, poor centration certainty, or poor segmentation certainty using TABS software were also excluded.^19^ These participants were excluded because of the well-recognized impact these factors have on retinal layer thickness.^20^

### Statistical Analysis

For this cross-sectional analysis, if both eyes of a participant were eligible for inclusion in the analysis, one eye was randomly selected using STATA software (version 16, StataCorp LP, College Station, TX, USA). The baseline characteristics of participants were described using mean (standard deviation [SD]) for continuous variables and number (percentage) for categorical variables. Diabetes status was analyzed in categories (Yes/ No). As mentioned earlier, DM was defined as those who had self-reported diabetes or self-reported use of insulin. We examined the associations of diabetes status with mRNFL, INL, GCIPL, photoreceptor layer, and total macular thickness using two multivariable linear regression models; model 1 adjusted for age and sex; and model 2 additionally adjusted for ethnicity, Townsend deprivation index, CVD, BMI, systolic blood pressure (SBP), antihypertensive medication, smoking status, refractive error, total cholesterol, HDL-C, and triglycerides. Participants were categorized into three groups: (1) participants with HbA1c <48 mmol/mol was subdivided into quintiles; (2) previously diagnosed diabetes; (3) undiagnosed diabetes: >48 mmol/mol and without diabetes diagnosis at enrollment.^21^ We then examined the association of people with no diabetes (normal HbA1c range in quintiles), previously diagnosed and undiagnosed diabetes with the retinal layer thicknesses, adjusted for the same covariables as above. HbA1c in the second quintile (32.1-34.0 mmol/mol) was used as the reference group because those with lower HbA1c levels, in people with or without diabetes consistently show greater mortality risks for reasons not well understood.^22^ In sensitivity analysis, IOP_cc_ was additionally adjusted for when mRNFL or GCIPL was the outcome variable, in view of its relationship with retinal ganglion cell health.^23^^,^^24^

## Results

**Figure 1.**
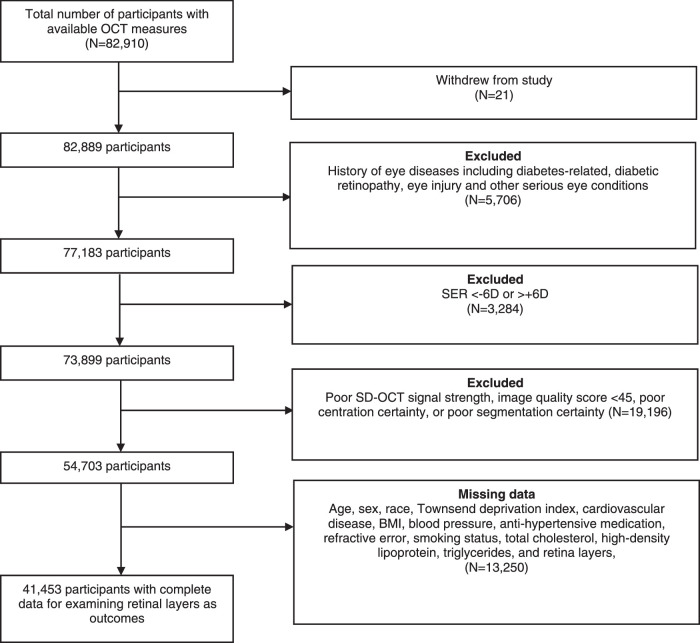
**Flowchart of participants included in the study**. BMI, body mass index; D, Diopters; SD-OCT, spectral-domain optical coherence tomography.

Of the 82,910 participants with available SD-OCT measures, 21 participants withdrew their consent. Of the 182,889, we excluded 28,186 participants according to the exclusion criteria (Fig. 1), leaving data on 54,703 participants, for whom complete data on covariates, including HbA1c, were available for 41,453 participants. The characteristics of participants with and without diabetes are shown in Table 1. The median (interquartile range [IQR]) of HbA1c in participants with and without diabetes were 48.0 mmol/mol (6.5%, IQR = 41.2–56.7 mmol/mol, 5.9–7.3%) and 34.9 mmol/mol (5.3%, IQR = 32.6–37.3 mmol/mol, 5.1–5.6%), respectively. Compared to participants without diabetes, those with diabetes were slightly older, more likely to be men, non-White, smokers, more likely to come from a more deprived area (less negative Townsend deprivation index), have higher BMI and triglycerides, more likely to have higher SBP, take antihypertensive medication, and have lower HDL-C and total cholesterol (see Table 1). In addition, we compared the characteristics of participants included to those excluded from the study. Given the very large sample size, even small differences between both groups were statistically significant. Compared to participants excluded from the study, those included were slightly younger (56.2 years versus 57.3 years; *P* < 0.001), more likely to be men (47.6% vs. 44.9%, *P* < 0.001), and White (92.6% vs. 91.3%, *P* < 0.001), had slightly lower BMI (27.19 kg/m^2^ vs. 27.26 kg/m^2^, *P* = 0.03), were more likely to be smokers (9.6% vs. 8.7%, *P* < 0.001), and had lower HbA1c measures (35.7 mmol/mol [5.4%] vs. 36.5 mmol/mol [5.5%], *P* < 0.001).

**Table 1.** Baseline Characteristics of Participants With and Without Diabetes

	Mean (SD) *n* (%)		
	No Diabetes	Diabetes	*P* Value
Number of participants	39,726	1727	
Age, y	56.1 (8.1)	59.1 (7.4)	<0.001
Sex			<0.001
Men	18,642 (46.9)	1073 (62.1)	
Women	21,084 (53.1)	654 (37.9)	
Ethnicity			<0.001
White	36,949 (93.0)	1429 (82.7)	
Non-White	2777 (7.0)	298 (17.3)	
Townsend Deprivation Index	−1.2 (2.9)	−0.5 (3.2)	<0.001
Smoking status			<0.001
Never	22,082 (55.6)	801 (46.4)	
Former	13,877 (34.9)	722 (41.8)	
Current	3767 (9.5)	204 (11.8)	
Body mass index (kg/m^2^)	27.0 (4.3)	30.5 (5.2)	<0.001
Antihypertensive medication			<0.001
No	35,912 (90.4)	1050 (60.8)	
Yes	3814 (9.6)	677 (39.2)	
Systolic blood pressure (mm Hg)	138.2 (19.5)	145.3 (19.2)	<0.001
Diastolic blood pressure (mm Hg)	81.8 (10.0)	81.3 (9.5)	0.024
Total cholesterol (mmol/L)	5.7 (1.1)	4.5 (1.1)	<0.001
HDL cholesterol (mmol/L)	1.5 (0.4)	1.2 (0.3)	<0.001
Triglycerides (mmol/L)	1.7 (1.0)	2.0 (1.1)	<0.001
**Ocular factors**			
Spherical equivalent refraction (D)	0.004 (2.0)	−0.003 (2.0)	0.88
Retinal nerve fiber layer thickness (µm)	29.1 (5.1)	28.0 (5.2)	<0.001
Ganglion cell inner plexiform layer thickness (µm)	73.3(6.2)	72.5 (6.4)	<0.001
Inner nuclear layer thickness (µm)	32.6 (2.2)	32.4 (2.2)	<0.001
Photoreceptor layer thickness (µm)	142.3 (7.5)	140.4 (7.9)	<0.001
Total macular thickness (µm)	277.3 (13.2)	273.3 (14.2)	<0.001

Mean (SD) for continuous variables and percentages for categorical variables.

D, Diopters; SD, standard deviation; HDL, high-density lipoprotein.

Table 2 shows the associations between diabetes status and thickness of the retinal layers. After adjusting for age and sex in model 1, compared to participants without diabetes, thinner retinal sublayers and total macular thickness were observed in participants with diabetes. In model 2, participants with diabetes had a thinner mRNFL, photoreceptor layer, and total macular layer, even after the additional adjustment for demographic, lifestyle factors, cardiovascular risk factors, and refractive error. Associations between diabetes status and GCIPL/INL thicknesses became nonsignificant.

**Table 2.** Multivariable Associations of Diabetes Status With Retinal Layer Thicknesses (*n* = 41,503)

		Diabetes					
		Model 1			Model 2		
	No Diabetes	*β*	95% CI	*P* Value	*β*	95% CI	*P* Value
Retinal nerve fiber layer	Ref	−0.89	(−1.14 to −0.64)	**<0.001**	−0.50	(−0.75 to −0.24)	**<0.001**
Ganglion cell-inner plexiform layer	Ref	−0.43	(−0.73 to −0.13)	**0.005**	−0.06	(−0.37 to 0.24)	0.68
Inner nuclear layer	Ref	−0.20	(−0.30 to −0.09)	**<0.001**	−0.02	(−0.13 to 0.09)	0.71
Photoreceptor layer	Ref	−2.09	(−2.45 to −1.73)	**<0.001**	−0.77	(−1.14 to −0.40)	**<0.001**
Total macular thickness	Ref	−3.62	(−4.25 to −2.99)	**<0.001**	−1.36	(−2.01 to −0.72)	**<0.001**

Model 1 adjusted for age and sex.

Model 2 adjusted for age, sex, ethnicity, Townsend Deprivation Index, cardiovascular disease, body mass index, systolic blood pressure, antihypertensive medication, smoking status, spherical equivalent refraction, total cholesterol, high-density lipoprotein cholesterol, and triglycerides.

Bold values denote statistical significance at the *P* < 0.05 level.

CI, confidence interval.

**Figure 2.**
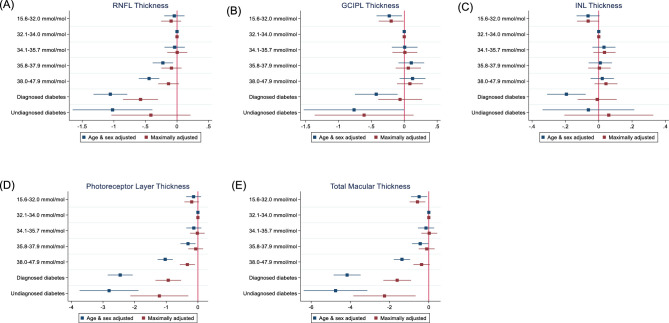
Adjusted beta coefficients of (**A**) retinal nerve fiber layer (RNFL) thickness, (**B**) ganglion cell-inner plexiform layer (GCIPL) thickness, (**C**) inner nuclear layer (INL) thickness, (**D**) photoreceptor layer thickness, and (**E**) total macular thickness by quintiles of baseline HbA1c in those without baseline diabetes (*n* = 39,475) and in participants with diagnosed diabetes (*n* = 1766) or undiagnosed diabetes (*n* = 262). Reference group (32.1-34.0 mmol/mol). Maximally adjusted for age, sex, ethnicity, Townsend deprivation index, cardiovascular disease, body mass index, systolic blood pressure, antihypertensive medication, smoking status, spherical equivalent refraction, total cholesterol, and high-density lipoprotein cholesterol and triglycerides. RNFL, retinal nerve fiber layer; GCIPL, ganglion cell inner plexiform layer; INL, inner plexiform layer.

Figure 2 examines the multivariable associations of quintiles of HbA1c across the range from normal to prediabetes, diagnosed, and undiagnosed diabetes with the thickness of the retinal layers. Compared to the second HbA1c quintile, participants in the fifth HbA1c quintile and those with diagnosed and undiagnosed diabetes had thinner mRNFL, photoreceptor layer, and total macular thicknesses after adjusting for age and sex. After adjusting for all covariables, the associations of quintiles of HbA1c, diagnosed, and undiagnosed diabetes with retinal layers were attenuated. Compared to the second HbA1c quintile, those in the fifth HbA1c quintile, diagnosed, and undiagnosed diabetes had reduced photoreceptor layer thickness (β = −0.33 µm, 95% confidence interval [CI] = −0.57 to −0.09, *P* = 0.006, β = −0.94 µm, 95% CI = −1.36 to −0.53; *P* < 0.001, and β = −1.22 µm, 95% CI = −2.13 to −0.30, *P* = 0.009, respectively). Participants with diagnosed and undiagnosed diabetes and those in the first HbA1c quintile had thinner total macular thickness (β = −1.61 µm, 95% CI = −2.31 to −0.91, *P* < 0.001, β = −2.26 µm, 95% CI = −3.85 to −0.67, *P* = 0.005, and β = −0.58 µm, 95% CI = −0.98 to −0.18, *P* = 0.004, respectively). Reduced RNFL thickness (β = −0.58 µm, 95% CI = −0.85 to −0.30, *P* < 0.001) were observed in participants with diagnosed diabetes.

## Discussion

In this large study of UK Biobank participants with and without diabetes, the most striking novel finding was the association of higher levels of HbA1c in the normal range (<48.0 mmol/mol) with a thinner photoreceptor layer. In addition, we confirmed associations observed in previous studies and showed that participants with diabetes were more likely to have thinner mRNFL, photoreceptor layer, and total macular thickness compared to those without diabetes, with much of the difference in total macular thickness and photoreceptor layer being driven by factors other than higher HbA1c given the substantial attenuation of associations after adjustment for potential confounding factors. Furthermore, our results show that glycaemia levels below the current diabetes diagnostic threshold are associated with a thinner photoreceptor layer. To our knowledge, this is the first adequately powered study to report associations of retinal layer thickness and HbA1c in both the normal and diabetic range. This finding is in keeping with previous research highlighting HbA1c a continuous risk factor for mortality through the whole population distribution.^25^ In contrast, the relationship between HbA1c and stroke risk appears to be more consistent with a threshold relationship.^26^

Our study showed a thinner mRNFL, photoreceptor layer, and total macular thickness in people with diabetes, but we did not observe statistically significant differences in the GCIPL or INL thickness between people with and without diabetes. Conversely, previous studies showed reduced thickness of the retinal sublayers and total macular thickness in people with diabetes compared to healthy controls. Van Dijk et al. reported thinner OCT derived thickness of the mRNFL and GCIPL in patients with diabetes compared with healthy controls,^27^^,^^28^ whereas Sohn et al. reported that mRNFL was thinner in the eyes of six donor eyes with DM than in the eyes of six control eyes.^29^ It should be noted that lack of change in other sublayer dimensions does not exclude the possibility of altered cellular function in diabetes.

Few studies have examined the association between HbA1c and retinal layer thickness, and the findings have been inconsistent. A negative association was previously observed between HbA1c levels and the temporal outer subfield of the total macular thickness,^30^ whereas Asefzadeh et al. did not identify a correlation between HbA1c levels and total macular thickness in 116 patients with diabetes.^31^ However, previous studies were of a much smaller sample size, which may affect the precision of the estimates. In addition, previous studies either did not adjust for any confounders^27^^,^^28^ or did not adjust for important confounders, including cardiovascular risk factors, including blood pressure, or relevant ocular factors.^27^^–^^29^ Hyperglycemia may stimulate the accumulation of inflammatory mediators and reactive oxidative species in the retina, resulting in the activation of microglial cells.^32^ Higher levels of HbA1c have been associated with an increase in hyper-reflective dots in SD-OCT imaging, which are thought to represent activated retinal microglial cells induced by inflammation in patients with diabetes.^33^ Studies have reported that of all the retinal cells, photoreceptor cells contributed the most oxidative stress and local inflammation in the retina of diabetic mice, suggesting that photoreceptor cells play an importance role in the initiation of DR.^34^ This might be a plausible explanation for our findings on the photoreceptor layer. Further studies are required to validate this finding.

After adjusting for demographic, cardiovascular risk factors, and ocular factors, the associations between HbA1c and diabetes status with retinal layer thickness were attenuated, especially for photoreceptor and total macular thickness. This suggests that lifestyle advice to maintain HbA1c level <48.0 mmol/mol, which improves blood pressure or lipid levels, may also be beneficial for the retinal health. Our study showed that participants in the first HbA1c quintile had thinner total macular thickness, compared to those in the second quintile. This association may be related to factor in such individuals that leads them to have a higher risk of mortality, as has been consistently demonstrated in multiple populations, as elegantly summarized by Rutter.^22^

This is the largest study to date, to our knowledge, that examined the association of HbA1c levels (across both the non-diabetes and diabetes range) with retinal layer thickness. Limitations of the study include a low response rate of 5.5% in UK Biobank, making participation analogous to a volunteer cohort. However, associations with disease outcomes should not be influenced by this fact. Participants are healthier and belong to a higher socioeconomic group than the average UK population. The status of diabetes obtained from a questionnaire and may be subject to misclassification bias. However, the SD-OCT measurements were all objective and because the participants were most likely unaware of their SD-OCT measures, it is most likely a nondifferential misclassification bias and will therefore skew the associations toward the null. Due to the strict exclusion criteria and quality control process that excluded participants, this could have led to selection bias. This may limit the generalizability of our results, but it is unlikely that the direction of associations on retinal layers would be affected by selection bias as the baseline characteristics of included versus excluded participants were only slightly different, and these variables were adjusted for in the multivariable models. Furthermore, that findings were broadly similar in those with undiagnosed diabetes based on elevated HbA1c, adds confidence that the results in those with diabetes are likely robust. It is noteworthy that the cross-sectional nature of this study limits the ability to determine the causality and temporality inferences. Our results were obtained using data from a middle-aged UK population, where age-related retinal neurodegeneration could potentially interfere with our findings. Further longitudinal studies with matched healthy controls are warranted to address this concern. Finally, despite HbA1c being an objective measurement, it has limitations in representing the overall, long-term glucose control because HbA1c levels only reflect an average glucose metabolism in the past 3 months.

In conclusion, we identified associations of thinner mRNFL, photoreceptor layer, and total macular thickness in people with diabetes of an older middle-aged UK population. Although the glycemia-related changes were absent or minimal for most of the retinal layers, we discovered a novel, albeit modest, association between higher HbA1c levels and a thinner photoreceptor layer in the normal range. Our results add to the growing evidence of early retinal neurodegeneration in people with diabetes, and in people whose HbA1c levels are below the current diabetes diagnostic threshold.

## Supplementary Material

Supplement 1
